# NOD1 and NOD2 Signaling in Infection and Inflammation

**DOI:** 10.3389/fimmu.2012.00328

**Published:** 2012-11-08

**Authors:** Lilian O. Moreira, Dario S. Zamboni

**Affiliations:** ^1^Faculdade de Farmácia, Departamento de Análises Clínicas e Toxicológicas, Universidade Federal do Rio de JaneiroRio de Janeiro, Brazil; ^2^Faculdade de Medicina de Ribeirão Preto, Departamento de Biologia Celular, Molecular e Bioagentes Patogênicos, Universidade de São PauloRibeirão Preto, Brazil

**Keywords:** NOD1, NOD2, RIPK2, intracellular pathogens, innate immunity

## Abstract

Sensing intracellular pathogens is a process mediated by innate immune cells that is crucial for the induction of inflammatory processes and effective adaptive immune responses against pathogenic microbes. NOD-like receptors (NLRs) comprise a family of intracellular pattern recognition receptors that are important for the recognition of damage and microbial-associated molecular patterns. NOD1 and NOD2 are specialized NLRs that participate in the recognition of a subset of pathogenic microorganisms that are able to invade and multiply intracellularly. Once activated, these molecules trigger intracellular signaling pathways that lead to the activation of transcriptional responses culminating in the expression of a subset of inflammatory genes. In this review, we will focus on the role of NOD1 and NOD2 in the recognition and response to intracellular pathogens, including Gram-positive and Gram-negative bacteria, and on their ability to signal in response to non-peptidoglycan-containing pathogens, such as viruses and protozoan parasites.

## Introduction

The immune system is able to recognize a large variety of microorganisms and their molecules through different receptors expressed by innate immune cells, such as macrophages, neutrophils, NK cells, and dendritic cells. The initial recognition of pathogenic microorganisms is critical for the generation of an appropriate acquired immune response. This process occurs through the interaction of microbial- or damage-associated molecular patterns (MAMPs or DAMPs, respectively) with the specific pattern recognition receptors (PRRs) present on the host cell surface or in the cytosolic compartment. Among the PRRs, the Toll-like receptors (TLRs), NOD-like receptors (NLRs), RIG-like helicases (RLRs), and AIM2-like receptors (ALRs) have been extensively investigated and play important roles as the major PRRs, forming the first line of defense against infectious agents (reviewed in Kawai and Akira, 2009; Schroder and Tschopp, 2010; Bonardi et al., 2012).

The TLRs are a group of transmembrane receptors that are able to recognize a large variety of MAMPs from different pathogenic microorganisms and induce the activation of the innate immune system (Janeway and Medzhitov, 2002). In humans, 10 functional TLRs have been identified, and a large amount of data demonstrates that TLRs may also work as sensors for self/endogenous molecules or “alarmins” that contribute to inflammatory processes and may be important for the maintenance of host homeostasis (reviewed in Kawai and Akira, 2009).

About 15 years ago, another family of PRRs was identified in humans. Proteins from this family contain a nucleotide-binding and oligomerization domain (NOD) and were named NLRs (Inohara et al., 1999, 2000; Girardin et al., 2001; Hoffman et al., 2001; Albrecht et al., 2003). The NLR family includes 22 members identified in humans and more than 30 in mice (Ting et al., 2008; Schroder and Tschopp, 2010).

The most studied NLRs belong to the NLR*C* and NLR*P* subgroups. The former is composed of receptors containing a *P*yrin domain in the amino-terminal region of the protein; these receptors are usually involved in the activation of caspase-1 and the assembly of the inflammasome, a molecular platform that has been reviewed elsewhere (Kanneganti et al., 2007; Schroder and Tschopp, 2010; Shaw et al., 2010). The NLR*C* subgroup is composed of receptors containing a *C*ard domain in the amino-terminal region and includes members such as NOD1 and NOD2 that play important roles in pathogen recognition and the activation of immune responses. NOD1 and NOD2 are encoded by the *card4* and *card15* genes, respectively, and are involved with the recognition of peptidoglycan moieties from Gram-positive and Gram-negative bacteria (Inohara et al., 2001; Chamaillard et al., 2003; Girardin et al., 2003a,b,c). Nevertheless, increasing numbers of recent reports suggest that NOD1 and NOD2 have important functions in non-bacterial infections. Whether NOD1 and NOD2 sense other structures and microbes or participate only as signaling partners is still unclear.

In this review, we will focus on functional aspects of the NOD1 and NOD2 proteins and discuss recent findings related to their roles in microbial recognition and the induction of inflammatory responses that lead to the restriction of infections with bacterial and non-bacterial pathogenic microbes.

## Structure and Signaling of NOD1 and NOD2

Structurally, NLRs are multi-domain proteins that contain an N-terminal Caspase Recruitment Domain (CARD) that associates with downstream signaling molecules, a centrally located nucleotide-binding oligomerization domain (NBD or NACHT), and a C-terminal leucine-rich repeat domain (LRR) or sensor domain (Proell et al., 2008; Schroder and Tschopp, 2010). NLRs vary in their N-terminal effector domains (PYD, CARD, BIR, and unclassified). Based on the domains present in this region, the NLRs are classified in two subgroups: NLR*C*s (*C*ARD), and NLR*P*s (*P*YRIN). The NLR members NOD1 and NOD2 belong to the NLRC subgroup as they contain an amino-terminal CARD domain and share the two common domains (NBD and LRR). NOD1 contains a single CARD domain, whereas NOD2 has two (Ogura et al., 2001). The carboxy terminal LRR domain is predicted to mediate protein–protein interactions and function as the regulatory domain. NOD1 and NOD2 contain multiple LRRs, a motif that has been linked to resistance to infection and is found in TLRs and plant R proteins (reviewed by Murray, 2005).

The idea that NOD1 and NOD2 function as intracellular receptors for bacterial peptidoglycan fragments emerged from studies using the over-expression of NOD1 and NOD2 and an NF-κB reporter in HEK293T cells (Inohara et al., 2001; Chamaillard et al., 2003; Girardin et al., 2003a,b,c). Further studies demonstrated that NOD1 activity was triggered by d-glutamyl-meso-diaminopimelic acid (DAP), which is found in Gram-negative and a few Gram-positive bacteria, including *Listeria* and *Bacillus* (Chamaillard et al., 2003; Hasegawa et al., 2006). In contrast, NOD2 activation was triggered by muramyl dipeptide (MDP), a peptidoglycan motif widely distributed among both Gram-positive and Gram-negative bacteria (Girardin et al., 2003b,c).

Until recently, the direct binding of NOD1 and NOD2 to their respective ligands, DAP and MDP, had not been demonstrated in a physiological milieu. However, the direct binding of MDP to NOD2 has recently been reported, suggesting the first biochemical evidence for a direct interaction between NOD2 and MDP (Grimes et al., 2012). In addition to NOD2 activation, different groups have reported that MDP is involved in the activation of other NLRs, including NLRP3 (Martinon et al., 2004; Pan et al., 2007) and NLRP1 (Hsu et al., 2008). The putative activation of NLRP3 and NLRP1 by MDP leads to the production and secretion of IL-1β, an important proinflammatory cytokine (Martinon et al., 2004; Hsu et al., 2008). Although it has been demonstrated that MDP triggers the production of cytokines, chemokines, nitric oxide (NO), and reactive oxygen species, several studies have shown that MDP alone is only weakly immunostimulatory (Parant et al., 1995; Wolfert et al., 2002; Pauleau and Murray, 2003; Kobayashi et al., 2005; Uehori et al., 2005; Kinsner et al., 2006; Moreira et al., 2008a). MDP has been shown to act synergistically with TLRs; the addition of MDP in combination with TLR agonists, such as lipoteicoic acid (LTA), LPS, and peptidoglycan, triggers a robust inflammatory response, including the release of proinflammatory cytokines such as IL-1β and IL-6 (Wolfert et al., 2002; Kim et al., 2007; Natsuka et al., 2008). As expected, the synergistic effect of MDP with TLR agonists is dependent on NOD2, but the molecular mechanisms responsible for this phenomenon are still not known. It is possible that TLR stimulation facilitates the internalization of MDP, a process that is required for NOD2 activation under physiological conditions.

Although the biological roles of DAP and MDP in the activation of NOD1 and NOD2 have been described, the mechanism underlying their internalization to the cytosol remains poorly understood. Recent studies using an HEK293 transfection system demonstrated that DAP and MDP reach the cytoplasm by endocytosis, in a clathrin-dependent manner. Moreover, the cytosolic internalization of the ligands was pH-dependent and occurred prior to the acidification mediated by the vacuolar ATPase (Lee et al., 2009). However, it remains to be determined whether this process also occurs in primary cells such as macrophages, which do not show robust activation in response to DAP or MDP alone (Parant et al., 1995; Wolfert et al., 2002; Pauleau and Murray, 2003; Kobayashi et al., 2005; Uehori et al., 2005; Kinsner et al., 2006; Moreira et al., 2008a).

**Figure 1:**
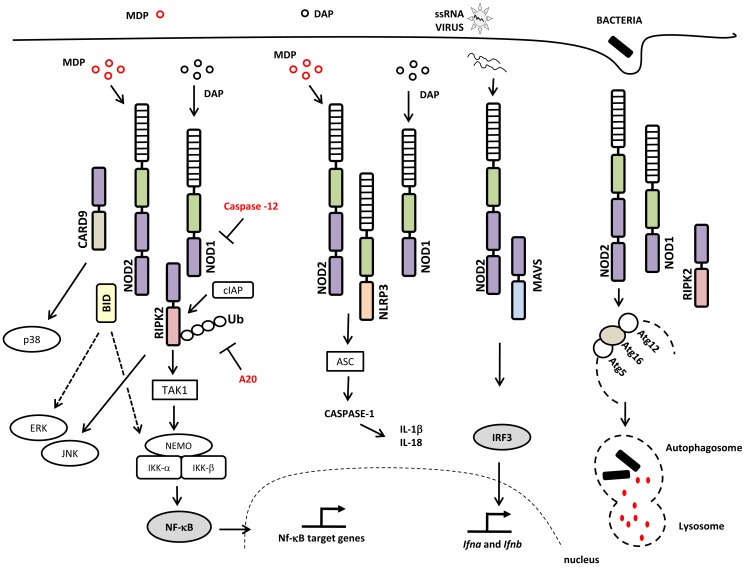
**NOD1 and NOD2 signaling pathways and interaction partners**. The canonical adaptor protein required for the activation of the signaling pathways downstream of NOD1 and NOD2 is the CARD-containing kinase RIPK2, a protein that interacts with NOD1 or NOD2 via homotypic CARD–CARD interactions leading to the activation of NF-κB and MAPKs. NOD/RIPK2 signaling can be inhibited by caspase-12 and/or A20. NOD1 and NOD2 proteins may also interact with the NLRP3 inflammasome, which leads to caspase-1 activation and IL-18 and IL-1β production. Viral ssRNA triggers a signaling pathway that is dependent on NOD2 and MAVS, leading to the activation of IRF3 and the induction of type I interferon. NOD1 and NOD2 activate the autophagy machinery through their interactions with ATG16L1 protein. Abbreviations include: NOD1, nucleotide-binding oligomerization domain 1; NOD2, nucleotide-binding oligomerization domain 2; RIPK2, receptor-interacting serine/threonine-protein kinase 2; CARD, caspase recruitment domain; JNK, c-Jun N-terminal kinases; TAK1, transforming growth factor-beta-activated kinase 1 TAK1; cIAP, inhibitor of apoptosis protein; NEMO, NF-κB essential modulator; IKK-γ, inhibitor of nuclear factor kappa-B kinase-gamma; IKK, I-kappa-B kinase; Ub, ubiquitinated; A20, ubiquitin-modified enzyme; ERK, extracellular-signal-regulated kinases; ASC, apoptosis-associated speck-like protein containing a CARD; IL-1β, interleukin-1; IL-18, interleukin-18; ssRNA, single-stranded RNA; MAVS, mitochondria anti-virus signaling protein; IFR3, interferon regulatory factor 3; Atg, autophagy-related gene; MAPK, mitogen-activated protein kinase; NF-κB, nuclear factor κB. Specific Domains of NOD1 and NOD2 are indicated: NBD or NACHT (green rectangle); LRR (hatched rectangle); CARD (purple rectangle).

The current model of NLR signaling proposes that upon specific recognition of their ligands, the NBD domains oligomerize and initiate the recruitment of interacting proteins leading to the interaction of the CARD domain with the CARD-containing kinase RIPK2 (also called RIP2/RICK) through a homotypic CARD–CARD interaction (Kobayashi et al., 2002; Park et al., 2007; Nembrini et al., 2009). This is accomplished via the recruitment of the E3 ubiquitin ligase TRAF6 to RIPK2, followed by TRAF6 autoubiquitination, polyubiquitination of RIPK2, and the ubiquitination-dependent signaling and activation of the TAK1 complex. The activated TAK1 complex promotes the K63-type polyubiquitination of IKK-β, culminating in the degradation of the nuclear factor kappa-B (NF-κB) repressor IκB, the translocation of NF-κB to the nucleus, and the transcription of proinflammatory genes (Hasegawa et al., 2008; Figure 1). RIPK2 is critical for the induction of NF-κB activation by NOD1 and NOD2, although the molecular details concerning how the NOD/RIPK2 complex stimulates NF-κB activation are only partially understood. In addition to NF-κB, NOD1, and NOD2 can activate the p38, ERK, and JNK mitogen-activated protein kinases (MAPKs; Pauleau and Murray, 2003; Kobayashi et al., 2005; Park et al., 2007). In addition, it was recently demonstrated that members of the inhibitor of apoptosis protein (IAP) family of proteins, such as XIAP (Krieg et al., 2009), cIAP1, and cIAP2 (Bertrand et al., 2009), interact with RIPK2. Most important, it was demonstrated that cIAP1 and cIAP2 function as E3 ubiquitin ligases responsible for the polyubiquitination of RIPK2, a process that is essential for the induction of NF-κB activation by NOD1 and NOD2 (Bertrand et al., 2009; Figure 1).

In addition to the bona fide interaction with RIPK2, NOD1, and NOD2 have been reported to interact with other NLRs that are important for caspase-1 activation. NOD2 was shown to specifically and directly interact with NLRP1, NLRP3, and NLRP12, whereas NOD1 interacts only with NLRP3 (Hsu et al., 2008; Wagner et al., 2009).

## NOD1 and NOD2 Expression and Signaling Regulation

NOD1 is widely expressed in many cell types, whereas NOD2 has been found in macrophages (Ogura et al., 2001), dendritic cells (Tada et al., 2005), paneth cells (Ogura et al., 2003), keratinocytes (Voss et al., 2006), epithelial intestinal cells (Hisamatsu et al., 2003), lung epithelial cells (Uehara et al., 2007), oral epithelial cells (Uehara et al., 2008), and osteoblasts (Marriott et al., 2005). NOD1 and NOD2 expression can be induced by different stimuli, such as live and heat-killed bacteria (Pudla et al., 2011); TLR ligands, IFN-γ, and TNF-α (Rosenstiel et al., 2003; Kim et al., 2007, 2008).

Little is known about how the NOD1 and NOD2 signaling pathways are regulated and at which step(s) of the cascade regulation occurs. One study showed that the ubiquitination of RIPK2 induced by MDP appears to be regulated by A20, a ubiquitin-modifying enzyme. A20-deficient cells exhibit amplified responses to MDP, including increased RIPK2 ubiquitylation, prolonged NF-κB signaling, and increased production of proinflammatory cytokines. The same phenotype was observed *in vivo* when A20-deficient mice were stimulated with MDP (Hitotsumatsu et al., 2008). Another study showed that caspase-12, an enzyme that modulates caspase-1 activation, binds to RIPK2, displacing TRAF6 from the signaling complex, a process that leads to the inhibition of ubiquitin ligase activity and consequently the blunting of NF-κB activation (LeBlanc et al., 2008).

Interestingly, several studies have shown that NOD1 and NOD2 bind/interact with different intracellular molecules that may positively or negatively regulate their signaling pathway. These studies suggest the existence of a highly complex network of protein–protein interactions underlying the biological functions of NOD1 and NOD2 (Table 1).

**Table 1:** **Molecules reported to directly interact with NOD1 and/or NOD2**.

Molecule	NOD1	NOD2	Function/model	Reference
RIPK2	+	+	Activation of NF-κB and MAPK/mice and human	Inohara et al. (2000), Kobayashi et al. (2002) and reviewed in (Inohara et al., 2002)
NLRP1		+	Inflammasome signaling/mice and human*	Hsu et al. (2008), Wagner et al. (2009)
NLPR3		+	Inflammasome signaling/human*	Wagner et al. (2009)
NLRP12		+	Inflammasome signaling/human*	Wagner et al. (2009)
Erbin		+	Negative regulator of NOD2 signaling/human*	McDonald et al. (2005)
CENT-1β	+	+	Negative regulator of NOD2 signaling/human*	Yamamoto-Furusho et al. (2006)
Rac1GTPase		+	Negative regulator of NOD2 signaling/human*	Eitel et al. (2008)
RIG-1		+	Negative regulator of NOD2 signaling/human*	Morosky et al. (2011)
JNK-binding protein (JNKBP1)		+	Negative regulator of NOD2 signaling/mice and human*	Lecat et al. (2012)
GRIM-19		+	Positive regulator of NOD2 signaling/human*	Barnich et al. (2005)
CARD9		+	Positive regulator of NOD2 signaling/mice and human*	Hsu et al. (2007)
Vimentin		+	Positive regulator of NOD2 signaling/human*	Stevens et al. (2012)
BID	+	+	Signaling for activation of ERK and NF-κB/mice and human*	Yeretssian et al. (2011)
Atg16L1	+	+	Induction of autophagy/mice and human*	Travassos et al. (2010)
			Induction of autophagy/human*	Cooney et al. (2010)
			Regulation of autophagy/human*	Homer et al. (2012)

*Phenotypes detected in human cells and/or in mammalian cells transfected with human molecules.

Positive regulators of NOD1 and NOD2 signaling have also been described. A study showed that GRIM-19, a protein involved in cell death, binds to NOD2 and is required for NF-κB activation (Barnich et al., 2005). Other molecules, such as the CARD-containing adaptor protein 9 (CARD9), operate downstream of NOD2 to trigger the RIPK2-independent activation of p38 and JNK (Hsu et al., 2007; Figure 1).

The NOD1 and NOD2 proteins have been reported to interact with the apoptotic pathway through additional mechanisms, aside from the role of IAP proteins in the NOD1 and NOD2 signaling pathway (Bertrand et al., 2009; Krieg et al., 2009). Yeretssian et al. (2011) demonstrated that BID, a BLC2 family protein, interacts directly with NOD1, NOD2, and the IκB kinase (IKK) complex in a process that is important for NF-κB and ERK activation, suggesting a non-apoptotic role for BID (Yeretssian et al., 2011). This study used bone marrow-derived macrophages (BMDMs) from *Bid*^−/−^ mice, which failed to activate NF-κB and ERK and were unable to secrete inflammatory cytokines upon stimulation with NOD ligands. In addition, *Bid*^−/−^ mice were unable to trigger cytokine production *in vivo* after challenge with NOD ligands (Yeretssian et al., 2011). In contrast, a study performed by Nachbur and colleagues demonstrated that *Bid*-deficient mice had the same phenotype as wild-type mice upon stimulation with NOD ligands. Moreover, the activation of NF-κB and ERK were similar in both *Bid*^−/−^and wild-type mice, suggesting that BID is not essential for NOD signaling (Nachbur et al., 2012). It is possible that differences in the gut microbiota associated with the animals affect their responses to NOD ligands. Additional studies, including co-housing experiments, will be required to clarify the role of BID in NOD signaling and to determine whether NOD proteins interfere with BID-dependent apoptosis.

Negative regulators of NOD signaling may reduce NOD1 or NOD2 signaling by inhibiting their interaction with other molecules in the pathway. One example is ERBIN, an LRR-containing protein that binds to NOD2, inhibiting MDP-induced signaling (McDonald et al., 2005). NOD1 and NOD2 signaling may also be inhibited by Centaurin β-1 (CENTβ1), a GTPase-activating protein that is a member of the ADP-ribosylation family and colocalizes with NOD1 and NOD2 in the cytoplasm of intestinal epithelial cells. Over-expression of CENTβ1 inhibited NOD1- and NOD2-dependent NF-κB signaling (Yamamoto-Furusho et al., 2006). Rac1 GTPase and retinoic-acid induced gene-1 (RIG-I) have also been shown to interact with NOD2 and inhibit its signaling (Eitel et al., 2008; Morosky et al., 2011).

## NOD1 and NOD2 in Chronic Diseases

Studies have revealed that some individuals with familial Crohn’s disease, a chronic inflammatory condition of the gut, have mutations in the *Card15* gene encoding NOD2 (Hugot et al., 2001; Ogura et al., 2001), but not NOD1 (Zouali et al., 2003). The most common *Card15* mutation associated with Crohn’s disease is an L100fs frameshift insertion at nucleotide 3020 (3020insC) in the LRR region of NOD2 (Hugot et al., 2001). Although significant, only a small percentage of Crohn’s disease patients harbor this *Card15* mutation (Hugot et al., 2001; Ogura et al., 2001; Lesage et al., 2002). In addition, mutations in the NBD region (R334W, R334Q, and L469F) have been associated with another inflammatory disorder known as Blau syndrome (Miceli-Richard et al., 2001). However, it is still poorly understood how polymorphisms in the NOD2 LRR (L100fs) or NBD (R334W, R334Q, and L469F) domains contribute to Crohn’s disease and Blau syndrome, respectively. Some current ideas about NOD2 polymorphisms in Crohn’s disease patients were previously reviewed and discussed elsewhere (Schroder and Tschopp, 2010). NOD2 polymorphisms have also been associated with a variety of human inflammatory diseases, such as atopic eczema (Weidinger et al., 2005), arthritis (Joosten et al., 2008; Vieira et al., 2012), atopic dermatitis (Macaluso et al., 2007), sarcoidosis (Kanazawa et al., 2005), and possibly asthma (Hysi et al., 2005; Duan et al., 2010), and endometrial (Ashton et al., 2010) or prostate cancer (Kang et al., 2012). More recently, it was demonstrated that human NOD2 polymorphisms were also associated with increased susceptibility to infectious diseases, such as leprosy (Zhang et al., 2009; Berrington et al., 2010a), and tuberculosis (Austin et al., 2008; Azad et al., 2012).

## NOD1 and NOD2 Functions in the Response to Bacterial Infection

The initial studies regarding the functional properties of NOD1 and NOD2 as intracellular receptors for the recognition of peptidoglycan and live bacteria were performed using *in vitro* models. These studies showed that NOD1 senses a substantial variety of Gram-negative bacteria while NOD2 senses Gram-positive and Gram-negative bacteria (Table 2).

**Table 2:** **Functions of NOD1 and/or NOD2 in host responses to pathogenic microbes**.

Microrganism	Model/protein	Reference
**PROTOZOA**		
*Toxoplasma gondii*	Mouse/NOD2	Shaw et al. (2009)
*Trypanosoma cruzi*	Mouse/NOD1	Silva et al. (2010)
*Plasmodium berghei ANKA*	Mouse/NOD1 and NOD2	Finney et al. (2009)
**BACTERIA**		
*Bacillus anthracis*	Mouse/NOD2	Hsu et al. (2008)
	Mouse/NOD1 and NOD2	Loving et al. (2009)
*Borrelia burgdorferi*	Human cells and mouse/NOD2	Oosting et al. (2010)
*Burkholderia pseudomallei*	Human cell line and mouse/NOD2	Pudla et al. (2011)
*Campylobacter jejuni*	Human cell line/NOD1	Zilbauer et al. (2007)
*Chlamydophila pneumoniae*	Human cell line/NOD1	Opitz et al. (2005), Shimada et al. (2009)
*Chlamydia trachomatis* and *Chlamydia muridarum*	Human cell line and mouse/NOD1	Welter-Stahl et al. (2006)
*Clostridium difficile*	Mouse/NOD1	Hasegawa et al. (2011)
*Citrobacter rodentium*	Mouse/NOD2	Kim et al. (2011b)
*Escherichia coli* entero-invasive	Human cell line/NOD1	Kim et al. (2004)
*Haemophilus influenzae*	Mouse/NOD1	Zola et al. (2008)
*Helicobacter pylori*	Human cell line, mouse, and clinical study/NOD1	Watanabe et al. (2011)
*Helicobacter hepaticus*	Mouse/NOD2	Biswas et al. (2010), Grubman et al. (2010)
*Legionella pneumophila*	Human cell line and mouse/NOD1 and NOD2	Shin et al. (2008), Berrington et al. (2010b), Frutuoso et al. (2010)
*Listeria monocytogenes*	Human cell line/NOD1	Opitz et al. (2006)
*Mycobacterium tuberculosis*	Human cells/NOD2	Brooks et al. (2011)
	Mouse/NOD2	Divangahi et al. (2008)
*Porphyromonas gingivalis*	Human cells/NOD1 and NOD2	Uehara et al. (2008)
*Propionibacterium acne*	Human cells and clinical study/NOD1 and NOD2	Tanabe et al. (2006)
*Pseudomonas aeruginosa*	Human cell line and murine fibroblast/NOD1	Travassos et al. (2005)
*Salmonella enterica*	Mouse/NOD1	Le Bourhis et al. (2009)
	Mouse/NOD1 and NOD2	Geddes et al. (2010), Keestra et al. (2011)
*Shigella flexneri*	Human cell line/NOD1	Girardin et al. (2001)
*Streptococcus pneumoniae*	Human cell line/NOD 2	Opitz et al. (2004)
**VIRUS**		
Respiratory syncytial virus (RSV)	Mice/NOD2	Sabbah et al. (2009)
*Murine* norovirus-1	Mouse/NOD1 and NOD2	Kim et al. (2011a)

The role of NOD1 and NOD2 in detecting specific microbial products *in vitro* was further demonstrated in mutant mice through the targeted deletion of these genes. Initial studies on NOD2 function in mice were performed using *Nod2* knockout mice created through an insertion mutation in the first exon encoding the first part of the second CARD domain (Pauleau and Murray, 2003). Surprisingly, these mice did not show evident intestinal dysfunction, even though BMDMs obtained from these mice did not respond to MDP stimulation, thus confirming the loss-of-function of NOD2 (Pauleau and Murray, 2003). Later, two other NOD2 transgenic mice were engineered: one with an insertion causing a frameshift mutation in the final LRR domain, corresponding to one of the most frequent mutation observed in familial Crohn’s disease patients (Maeda et al., 2005), and another with a loss-of-function insertion in the *Card15* locus (Kobayashi et al., 2005). As reported by Pauleau and colleagues, the transgenic mice constructed by Maeda et al. and Kobayashi et al. show no intestinal pathology under normal housing conditions (Pauleau and Murray, 2003).

Although *Nod2^−/−^* mice did not show any inflammatory phenotype in the gut, several reports demonstrated their increased susceptibility to bacterial infections. For *Listeria monocytogenes* infection, NOD2 was shown to be important for restricting bacterial multiplication because *Nod2^−/−^* mice orally infected with *L. monocytogenes* showed decreased production of β-defensins and an increased bacterial burden (Kobayashi et al., 2005; Kim et al., 2008).

Similar features were observed in *Nod1^−/−^* mice infected with *Helicobacter pylori*; NOD1 was shown to be important for the recognition of peptidoglycan translocated from the bacterial cell to the host cell cytoplasm through the *cag* type IV secretion system. This feature accounts for NOD1-dependent responses that generate resistance against infection (Viala et al., 2004; Boughan et al., 2006).

In a pulmonary model of infection with *Chlamydophila pneumoniae*, NOD2, and RIPK2 were found to be critical for host responses; *Nod2*- and *Ripk2*-deficient mice infected with *C. pneumoniae* exhibited impaired production of nitric oxide and chemokine (C-X-C motif) ligand 1 production and delayed neutrophil recruitment to the lungs (Shimada et al., 2009). Defective recruitment of neutrophils to the intestines was also observed in *Nod1^−/−^* mice infected with *Clostridium difficile*, possibly due to the impairment of CXCL1 production. The increased mortality of the *Nod1^−/−^* mice was accompanied by impaired *C. difficile* clearance and increased bacterial translocation from the intestines to other organs, a process that resulted in elevated levels of microbiota-derived endotoxin and IL-1β in the sera of *Nod1^−/−^* mice (Hasegawa et al., 2011).

Some authors have reported that both NOD1 and NOD2 function in a synergistic fashion to tune the appropriate responses to certain pathogens. For example, NOD1 and NOD2 double-deficient mice showed a significant reduction in the production of inflammatory cytokines and an increase in the bacterial colonization of the mucosal tissue in a *Salmonella* model of colitis. These phenotypes were not observed in *Nod1^−/−^* or *Nod2^−/−^* single knockouts (Geddes et al., 2010). The same group further demonstrated that NOD1 and NOD2 were crucial for the induction of mucosal Th17 responses during the early stages of infection with *Citrobacter rodentium* and *S. enterica* Typhimurium (Geddes et al., 2011). Of note, this response was dependent on the intestinal microbiota, a concept that may flourish in this field within the next few years.

Cooperation between NOD1 and NOD2 was also reported in a murine model of *Bacillus anthracis*. Mice deficient for both NOD1 and NOD2 were more susceptible to lethal challenge with *B. anthracis* and produced lower levels of proinflammatory molecules when compared with single knockouts (Loving et al., 2009). A similar phenomenon was observed after pulmonary infection of mice with *Legionella pneumophila*, an intracellular bacterial pathogen that has been used as an excellent model for investigating bacterial recognition by innate immune receptors (Vance, 2010; Massis and Zamboni, 2011). NOD1 or NOD2 single knockout mice effectively restricted the replication of *L. pneumophila* in the lungs; in contrast, RIPK2-deficient mice were less efficient at clearing pulmonary bacteria (Frutuoso et al., 2010). Additional investigation using *Ripk2^−/−^* mice indicated that the NOD/RIPK2 pathway cooperates with TLR signaling to restrict bacterial growth in mouse lungs; RIPK2/MyD88 double-deficient mice were significantly more susceptible to *L. pneumophila* infection compared with MyD88 single knockout mice (Archer et al., 2010).

Another study performed with *L. pneumophila* indicated that NOD1 and NOD2 drive distinct inflammatory responses (Berrington et al., 2010b). *Nod1^−/−^* mice showed impaired bacterial clearance and neutrophil recruitment to the alveolar space and decreased production of proinflammatory cytokines when compared with wild-type mice. In contrast, increased levels of lung neutrophils and proinflammatory cytokine production were observed in *Nod2^−/−^* infected mice. However, at later stages of infection, both *Nod1^−/−^* and *Nod2^−/−^* mice produced significantly increased levels of proinflammatory cytokines in the lungs (Berrington et al., 2010b). Collectively, these studies indicate that although NOD1/NOD2/RIPK2 signaling is not critical for host resistance against *Legionella pneumophila*, both NOD1 and NOD2 participate in the recognition of these bacteria *in vivo* (Archer et al., 2010; Berrington et al., 2010b; Frutuoso et al., 2010).

Regardless of the roles of NOD1 and NOD2 in the immune response to certain microbes, for some bacterial pathogens, such as *Coxiella burnetii* and *Brucella abortus*, NOD1 and NOD2 play no role in restricting bacterial replication in murine models of infection (Benoit et al., 2009; Oliveira et al., 2012). This may also be the case for other pathogenic bacteria that bypass and/or are refractory to NOD1- and NOD2-mediated immunity.

## NOD1 and NOD2 in Response to Non-Peptidoglycan-Containing Pathogens

As reviewed so far, the role of NOD1 and NOD2 in sensing bacterial products has been intensively investigated. Moreover, the use of *Nod1^−/−^* and *Nod2^−/−^* mice supported the key role of these molecules in restricting bacterial infection (Table 2). However, recent reports indicate that NOD1 and NOD2 also play a role in host responses against protozoan parasite infections.

*Nod2^−/−^* mice failed to clear *Toxoplasma gondii* infection and succumbed at similar rates to *MyD88^−/−^* mice. NOD2 was shown to be involved in a T cell-intrinsic function rather than being active in macrophages and DCs (Shaw et al., 2009). Moreover, *Nod2^−/−^* mice failed to trigger IFN-γ production and to induce the differentiation of Th1 lymphocytes (Shaw et al., 2009). However, these observations were not corroborated by studies performed by three independent groups (Caetano et al., 2011). The reason for these discrepancies maybe related to variations in the bacterial microbiota present in the guts of the NOD2-deficient mice. Further studies, including those involving co-housing and the use of germ-free mice, will be required to address this issue. The T cell-intrinsic defect in *Nod2^−/−^* mice could explain why these mice are partially defective at generating antigen-specific antibodies even in the absence of NOD2 ligands (Moreira et al., 2008a). Nonetheless, the role of NOD2 signaling in CD4+ T cells requires additional investigation.

Studies performed with other intracellular protozoan parasites, such as *Plasmodium berghei* ANKA and *Trypanosoma cruzi*, indicated that NOD2 was not required for host protection against these parasites (Finney et al., 2009; Silva et al., 2010). In contrast, NOD1 was associated with host resistance against *T. cruzi* infection *in vivo* (Silva et al., 2010). BMDMs from *Nod1^−/−^* mice showed impaired induction of NF-κB-dependent products in response to infection and failed to restrict *T. cruzi* infection in the presence of IFN-γ. Despite normal cytokine levels in the sera, *Nod1^−/−^* mice were highly susceptible to *T. cruzi* infection in a similar manner to *MyD88^−/−^* and NO synthase 2 (iNOS)-deficient mice. This study indicated that NOD1-dependent responses accounted for host resistance to *T. cruzi* infection via cytokine-independent mechanisms (Silva et al., 2010).

Interestingly, in addition to the detection of bacteria and protozoa, NOD2 has an important role in virus recognition during experimental infection (Sabbah et al., 2009). NOD2, but not NOD1, was shown to facilitate IRF3 activation and the production of type I IFN in response to single-stranded RNA (ssRNA) or infection with respiratory syncytial virus (RSV; Sabbah et al., 2009). The authors showed that ssRNA and RSV, which does not contain peptidoglycan, activated NOD2 through a mechanism that was dependent on MAVS (mitochondrial antiviral signaling protein) and led to the activation of IRF3. Another recent study demonstrated that bacteria-infected mice co-stimulated with poly I:C or IFN-γ or co-infected with murine norovirus-1 dramatically augmented NOD1 and NOD2 signaling and expression and the production of proinflammatory cytokines. This response was attenuated in NOD1/NOD2 double knockout or RIPK2-deficient mice. The crosstalk between NOD1/NOD2 and type I IFN signaling may somehow facilitate bacterial recognition; however, it also induces harmful effects in the virally infected host (Kim et al., 2011a). These data contribute to our understanding of the lethal effects of host co-infection by two pathogens that are not normally lethal in singly infected hosts (Jamieson et al., 2010).

Although these initial reports indicated that NOD1 and NOD2 might be important in responses to non-bacterial pathogens, further studies will be required to address the roles of NOD1 and NOD2 in the recognition of pathogens that lack peptidoglycan moieties. In this context, it is important to determine whether NOD1 and NOD2 act as bonafide receptors for these pathogens or whether they take part in signaling pathways triggered by other innate immune molecules.

## NOD1 and NOD2 in Autophagy Induction

Recent reports have shown that NOD1 and NOD2 are associated with the induction of macroautophagy, a highly conserved degradation system in which specific cell components, including damaged organelles or proteins, are engulfed into a double-layered membrane structure for further degradation (reviewed in Lu and Walsh, 2012). Autophagy is also considered an immunologically regulated process and represents an innate defense mechanism that can control the replication of intracellular pathogens, including *Mycobacterium tuberculosis* and others (Gutierrez et al., 2004; Ogawa et al., 2011). Both NOD1 and NOD2 were shown to recruit ATG16L1 to the plasma membrane at the site of bacterial entrance to initiate autophagy (Travassos et al., 2010). In addition, NOD2 agonists induced autophagy in DCs in a RIPK2-, ATG5-, and ATG7-dependent manner (Cooney et al., 2010). In fact, polymorphisms in the ATG16L1 gene are known to be a risk factor for the development of Crohn’s disease in humans (Naser et al., 2012). NOD-dependent autophagy induction occurs after cell stimulation with peptidoglycan or live *Shigella flexneri* and is independent of RIPK2 (Travassos et al., 2010). Nonetheless, additional studies have reported that RIPK2 is necessary for the NOD2-mediated induction of autophagy (Cooney et al., 2010; Anand et al., 2011; Homer et al., 2012). The studies performed by Travassos et al. and Cooney et al. were the first to suggest that NOD1 and NOD2 function as a molecular scaffold for the autophagy machinery and may thereby act as nucleation sites for autophagy initiation (Cooney et al., 2010; Travassos et al., 2010).

More recently, different groups have reported a role for NOD1 and/or NOD2 in the induction of autophagy in response to several pathogens including *S. Typhimurium*, *M. tuberculosis*, adherent-invasive *E. coli*, and *L. monocytogenes* (Anand et al., 2011; Homer et al., 2012; Juarez et al., 2012; Lapaquette et al., 2012).

## NOD1 and NOD2 in Adaptive Immunity

Although NOD1 and NOD2 are associated with innate immune responses, several reports have demonstrated their involvement in the induction of adaptive immune responses. In fact, for certain infections, impairment of NOD1 and NOD2 function interferes with both innate and adaptive immune responses (Divangahi et al., 2008; Shaw et al., 2009).

NOD2 stimulation with MDP triggers an antigen-specific immune response with a Th2-type polarization profile, characterized by the production of IL-4 and IL-5 by T cells and IgG1 antibody responses (Magalhaes et al., 2008). Other studies have suggested that NOD1 is important for T cell priming and antibody production. NOD1 stimulation with its agonist alone was sufficient to drive a Th2 antigen-specific immune polarization. NOD1-deficient mice showed a reduced frequency of IFN-γ − producingCD4^+^ and CD8^+^ T cells and decreased antibody production, suggesting that NOD1 was required for optimal T and B cell responses (Fritz et al., 2007); a similar effect was also described for NOD2 (Shaw et al., 2009).

NOD2 was also found to be critical for the induction of both Th1- and Th2-type responses following co-stimulation with TLR agonists (Magalhaes et al., 2008). The lack of NOD2-dependent Th2 differentiation in a subset of Crohn’s disease patients might explain how the Th1-mediated inflammation at the intestinal mucosa contributes to the pathogenesis of the disease (Magalhaes et al., 2008). However, the use of MDP as an adjuvant is controversial because MDP alone is a weak adjuvant compared with TLR agonists (Magalhaes et al., 2008). Therefore, MDP may be inefficient at triggering adequate adaptive immune responses, as previously reported (Moreira et al., 2008a).

## Regulation of Immune Responses by NOD1 and NOD2

It is possible that NOD2 has a regulatory function for innate immune responses, acting as a transducer modifier as previously suggested (Murray, 2005). Watanabe and colleagues demonstrated that mixed splenocyte cultures from *Nod2^−/−^* mice produced high levels of IL-12 upon stimulation with PGN, and a similar phenotype was observed *in vivo* when *Nod2^−/−^* mice were injected with PGN (Watanabe et al., 2004). Intact NOD2 signaling inhibited the TLR2-driven activation of NF-κB, particularly the NF-κB subunit c-Rel. Moreover, NOD2 deficiency or the presence of a Crohn’s disease-like *Card15* mutation increased the TLR2-mediated activation of NF-κBc-Rel and Th1 responses (Watanabe et al., 2004). Thus, *Card15* mutations may lead to disease by causing excessive Th1 responses. This finding was corroborated by the fact that BMDMs from *Nod2^−/−^* mice produced less IL-10 upon stimulation with PGN purified from *Streptococcus pneumoniae*, suggesting that NOD2 may have a regulatory effect on IL-10 production (Moreira et al., 2008b). The reduction of IL-10 production would lead to increased production of IL-12, thereby contributing to the excessive inflammation observed in Crohn’s disease patients (Moreira et al., 2008b). In fact, it was recently demonstrated that the Crohn’s-disease-associated NOD2 mutation suppresses human IL-10 transcription by inhibiting the activity of the nuclear ribonucleoprotein hnRNP-A1 (Noguchi et al., 2009). The NOD2 3020insC mutation blocks p38 phosphorylation of hnRNP-A1, which impairs hnRNP-A1 binding to the IL-10 locus in peripheral blood mononuclear cells from Crohn’s disease patients (Noguchi et al., 2009). These findings are consistent with the previous suggestion that NOD2 may interfere with the production of IL-10 (Moreira et al., 2008b), a cytokine that is important for the regulation of inflammatory processes.

In another mouse model, a NOD2 mutation potentiated NF-κB activity and IL-1β processing, suggesting that NOD2 may act as a positive regulator of NF-κB activation and IL-1β secretion, thereby increasing the intestinal inflammation observed in Crohn’s disease patients (Maeda et al., 2005). The mechanism by which mutations in the *Card15* gene influence the chronic inflammation status observed with Crohn’s disease is still poorly understood. Because signaling via mutated NOD2 proteins leads to defective activation of NF-κB, one hypothesis is that mutations causing deficient NF-κB-dependent Th1 responses increase susceptibility to gut infections. This idea is supported by recent findings showing that wild-type NOD2, but not the mutant variants found in humans, can mediate autophagy, thereby allowing the clearance of bacterial pathogens that reach the host cell cytoplasm.

## Concluding Remarks

The NOD1 and NOD2 proteins play a remarkable role in host immune responses. Despite their undisputed importance for host defense, the specific mechanisms underlying their functions are yet to be determined. Although it is clear that these molecules are able to sense bacterial cell wall components and pathogens, their unique role as intracellular “receptors” is still a matter of debate. Although direct binding of MDP to NOD2 has recently been demonstrated (Grimes et al., 2012), alternative functions for the NODs, such as regulation of signal transduction systems have been proposed (Murray, 2005; Strober et al., 2006), thus corroborating the idea that NOD1 and NOD2 are multifaceted proteins. As mentioned in this review, NOD2 may interfere with IL-10 production and act as a regulatory molecule rather than an inflammatory inducer. The roles of NOD1 and NOD2 in the induction and resolution of inflammatory processes are largely unknown. A more comprehensive understanding of the functions of NOD1 and NOD2 in mammalian immunity may allow the use of new pharmacological interventions to either boost or reduce inflammatory responses against pathogenic microbes.

## Conflict of Interest Statement

The authors declare that the research was conducted in the absence of any commercial or financial relationships that could be construed as a potential conflict of interest.
